# Identification of epidermal growth factor receptor-derived peptides immunogenic for HLA-A2^+^ cancer patients

**DOI:** 10.1038/sj.bjc.6601728

**Published:** 2004-03-16

**Authors:** H Shomura, S Shichijo, S Matsueda, T Kawakami, Y Sato, S Todo, K Itoh

**Affiliations:** 1 Department of Immunology, Kurume University School of Medicine, 67 Asahi-machi, Kurume, Fukuoka 830-0011, Japan; 2 Department of General Surgery, Hokkaido University Graduate School of Medicine, N15 W7, Sapporo, Hokkaido 060-8788, Japan

**Keywords:** epidermal growth factor receptor, peptides, CTLs, Ab, cancer vaccine

## Abstract

Epidermal growth factor receptor (EGFR) is one of the most appropriate target molecules for cancer therapy because of its relatively high expression in about one-third of all epithelial cancers in correlation with neoplasmic progression. With respect to EGFR-targeted therapies, antibodies and tyrosine-kinase inhibitors have been intensively studied, a novel EGFR-tyrosine-kinase inhibitor ZD1839 has been approved as an anticancer drug, and many other agents are now under clinical trial. In addition, cytotoxic T lymphocyte (CTL)-directed epitope peptides could be another class of compounds useful in EGFR-targeted therapies. However, there is presently no information on CTL-directed peptides of EGFR. Therefore, from the viewpoint of development of peptide-based cancer therapy, this study was intended to determine the EGFR-derived peptides recognised by both cellular and humoral immunities in HLA-A2^+^ epithelial cancer patients. We herein report finding of two such types of EGFR-derived peptides at position 479–488 and 1138–1147, both of which were recognised by the majority of patients' sera (IgG), and also possessed the ability to induce HLA-A2-restricted peptide-specific CTLs against EGFR-positive tumour cells in peripheral blood mononuclear cells (PBMCs) of epithelial cancer patients. These results may provide a scientific basis for the development of EGFR-based immunotherapy for HLA-A2^+^ cancer patients.

Epithelial growth factor receptor (EGFR) plays an important role in epithelial biology and in many human malignancies (Coussens *et al*, 1985; Yamamoto *et al*, 1986; Salomon *et al*, 1995). A line of evidence that the EGFR plays a role in the pathogenesis of various cancers has led to the rational design and development of agents that selectively inhibit this receptor. Classes of compounds used in these EGFR-targeted therapies are mainly antibodies (Abs) and tyrosine-kinase inhibitors. Among them, ZD1839 (Iressa) is therapeutically effective for patients with advanced non-small-cell lung cancer (NSCLC) (Fukuoka *et al*, 2003; Miller *et al*, 2003). In addition, cytotoxic T lymphocyte (CTL)-directed epitopes could be another class of compound useful in EGFR-targeted therapies as peptide vaccines for cancer patients whose tumours overexpress EGFR. However, there is little information on CTL-directed epitopes of EGFR, although such CTL-directed peptides of HER2/neu, a family of EGFR, have been reported over the past decade (Fisk *et al*, 1995; Peoples *et al*, 1995; Kawashima *et al*, 1999; Okugawa *et al*, 2000). In previous clinical studies, we reported that some CTL-directed peptides from nonmutated proliferation-related proteins had the ability to elicit both cellular and humoral immune responses *in vivo* (Mine *et al*, 2003; Noguchi *et al*, 2003; Sato *et al*, 2003). Further, the levels of antipeptide Abs in postvaccination sera were well correlated with the overall survival of advanced lung cancer patients who received peptide vaccination (Mine *et al*, 2003). In addition, there is a line of evidence suggesting the existence of more highly immunogenic peptides that are capable of inducing both cellular and humoral immune responses (Disis *et al*, 1997). Therefore, to assist in the development of peptide-based cancer therapy, we here attempted to identify such peptides, and report the discovery of two peptides that can be considered as vaccine candidates for HLA-A2^+^ cancer patients.

## MATERIALS AND METHODS

### Samples and cell lines

After written informed consent was obtained, sera and peripheral blood mononuclear cells (PBMCs) were collected from NSCLC patients at Kurume University Hospital. Peripheral blood mononuclear cells and sera were also obtained from healthy donors (HDs). All subjects were free from human immunodeficiency virus (HIV) infection. All sera and PBMCs were cryopreserved at −80 and −196°C until use, respectively. The expression of HLA-class I antigens on these PBMCs was serologically defined by the conventional methods as reported previously (Noguchi *et al*, 2003), and HLA-A2 subtypes were determined by the sequence-specific oligonucleotide probe method as reported previously (Ito *et al*, 2001). The following tumour cell lines were used as target cells in a 6-h ^51^Cr-release assay in this study: 11–18 (HLA-A2/24, human lung adenocarcinoma, EGFR^+^), QG56 (HLA-A26, human lung squamous cell carcinoma, EGFR^+^), SKOV3 (HLA-A3/28, human ovarian cancer, EGFR^+^) and SKOV3-A2 (HLA-A2-transfected SKOV3). The expression of EGFR in these cell lines except 11-18 was already reported (Xu *et al*, 1999; Hasmann *et al*, 2003). The expression of EGFR in 11-18 tumour cells was checked by flow cytometric assay with an immunofluorescence-labelled anti-EGFR monoclonal antibody (mAb) (Santa Cruz Biotechnology, Santa Cruz, CA, USA) (Parkar *et al*, 2001), and it was also expressed in 11–18 tumour cells (data not shown). Phytohaemagglutinin (PHA)-blastoid T cells from PBMCs were also used as a negative control of target cells for a 6-h ^51^Cr-release assay. For peptide loading, T2 (HLA-A2, T-B hybridoma) cells were also used in this study.

### Peptides and quantification of antipeptide-specific IgG

The following peptides were purchased from BioSynthesis (Lewisville, TX, USA): 29 kinds of EGFR-derived peptides with HLA-A0201 and A0205 binding motifs at positions 10–18, 40–49, 61–70, 88–96, 110–118, 431–440, 479–488, 599–607, 653–662, 654–662, 656–664, 665–674, 681–689, 702–800, 717–725, 729–738, 765–776, 777–786, 791–799, 811–819, 813–822, 813–821, 843–851, 852–861, 944–952, 944–953, 945–953, 1001–1010, and 1138–1147, respectively. An HIV peptide with an HLA-A0201 binding motif (SLYNTVATL) was also provided as a negative control. Antipeptide-specific IgG levels in sera were measured by an enzyme-linked immunosorbent assay (ELISA) as reported previously (Sato *et al*, 2003). In brief, serum samples were serially diluted with 0.05% Tween 20-Block Ace (Yukijirushi Nyugyo, Tokyo, Japan), and 100 *μ*l well^−1^ of diluted serum was added to the peptide (20 *μ*g well^−1^)-immobilized Nunc Covalink plates (Roskilde, Denmark). Antipeptide Abs were detected with a rabbit anti-human IgG (*γ*-chain-specific) (DAKO, Glostrup, Denmark). For determining the limit of sensitivity of ELISA, sera from 11 HDs (HIV-negative) were measured for their reactivity to an HIV peptide by the assays, and the mean±2 standard deviations (s.d.) of optimal density (OD) at a serum dilution of 1 : 100 was 0.02±0.04. The mean+2 s.d. value (0.06) was then determined as the cutoff value. To test the specificity of antipeptide IgG in serum samples, 100 *μ*l well^−1^ of serum samples (100 times dilution with 0.05% Tween 20-Block Ace) was absorbed with immobilised peptides (20 *μ*g well^−1^) in wells of the plate for 2 h at 37°C. The absorption, followed by testing of the antipeptide IgG with ELISA, was repeated three times. To test the antipeptide IgG response to a whole molecule of EGFR, patients' sera possessing antipeptide activity were also absorbed with either immobilised human EGFR isolated from A431 cells with a purity of 85% (UPSTATE, Charlottesville, VA, USA) or immobilised human albumin as a negative control, followed by measurement of anti-peptide activity by ELISA.

To test the direct growth inhibition activity of antipeptide IgG, 11–18 tumour cells were cultured in the presence of three different concentrations of sera that had detectable levels of antipeptide activity. Namely, 11–18 cells at 1 × 10^3^ cells well^−1^ in a 96-well microculture plate (IWAKI, Chiba, Japan) were cultured for 12 h in the medium with 10% FCS followed by replacement of the culture medium to serum-free RPMI1640 with 1, 2, or 5% of serum possessing antipeptide activity. As controls, these sera absorbed with a corresponding peptide, sera without antipeptide activity from the two patients and two HDs were used. A total of 11–18 cells were also cultured in the RPMI1640 with 1, 2 and 5% FCS, respectively. After 24, 48 and 72 h incubation, the number of viable cells was determined by Cell Counting Kit-8 (Dojindo, Kumamoto, Japan). We also tested the antibody-dependent cell-mediated cytotoxicity of antipeptide IgG. Namely, the cytotoxicity of freshly-isolated PBMCs from HLA-A2^+^ HDs against T2 cell pulsed with an EGFR-derived peptide or an HIV peptide as a negative control was measured in the presence of heat-inactivated serum possessing antipeptide activity by a standard 6-h ^51^Cr-release assay. As controls, these sera absorbed with a corresponding peptide, sera possessing nonpeptide activity, and serum-free RPMI1640 medium were used. All sera used in the cytotoxicity assay were heat-inactivated at 56°C for 30 min.

### CTL induction

Peripheral blood mononuclear cells from HLA-A2^+^ epithelial cancer patients and HDs served as samples for the CTL induction assay. For induction of peptide-specific CTLs, PBMCs (15 × 10^4^ cells well^−1^) were incubated with 10 *μ*M of each peptide in four different wells of a 96-well microculture plate (Nunc) in 200 *μ*l culture medium containing interleukin-2 (IL-2), as reported previously (Mine *et al*, 2003). On the 14th day, the cells from each well were independently harvested, washed, and tested for their ability to produce interferon-*γ* (IFN-*γ*) in response to T2 cells pulsed with a corresponding peptide or a negative control peptide (HIV) in the duplicate assays. After an 18-h incubation, the supernatant was collected and measured for IFN-*γ* production by ELISA. Then the cells in the wells producing IFN-*γ* in response to a corresponding peptide were collected and further cultured with IL-2 alone for 10–14 days to obtain a large number of cells for a standard 6-h ^51^Cr-release assay against the various tumour cells described above. The method used for the ^51^Cr-release assay has been reported elsewhere (Mine *et al*, 2003). For an inhibition test, we used 20 *μ*g/ml of anti-HLA-class I (W6/32, IgG2a), anti-HLA-class II (H-DR-1, IgG2a), anti-CD4 (Nu-Th/i, IgG1), and anti-CD8 (Nu-Ts/c, IgG2a) mAbs. We also used an anti-CD14 (JML-H14, IgG2a) mAb as a negative control. For a competition assay to study the peptide specificity of the cytotoxicity, unlabelled T2 cells pulsed with the corresponding peptide or an HIV peptide as a negative control were added to the ^51^Cr-release assay at a cold-to-hot target cell ratio of 10 : 1. A two-tailed Student's *t*-test was employed for the statistical analysis in this study.

## RESULTS

**Figure 1:**
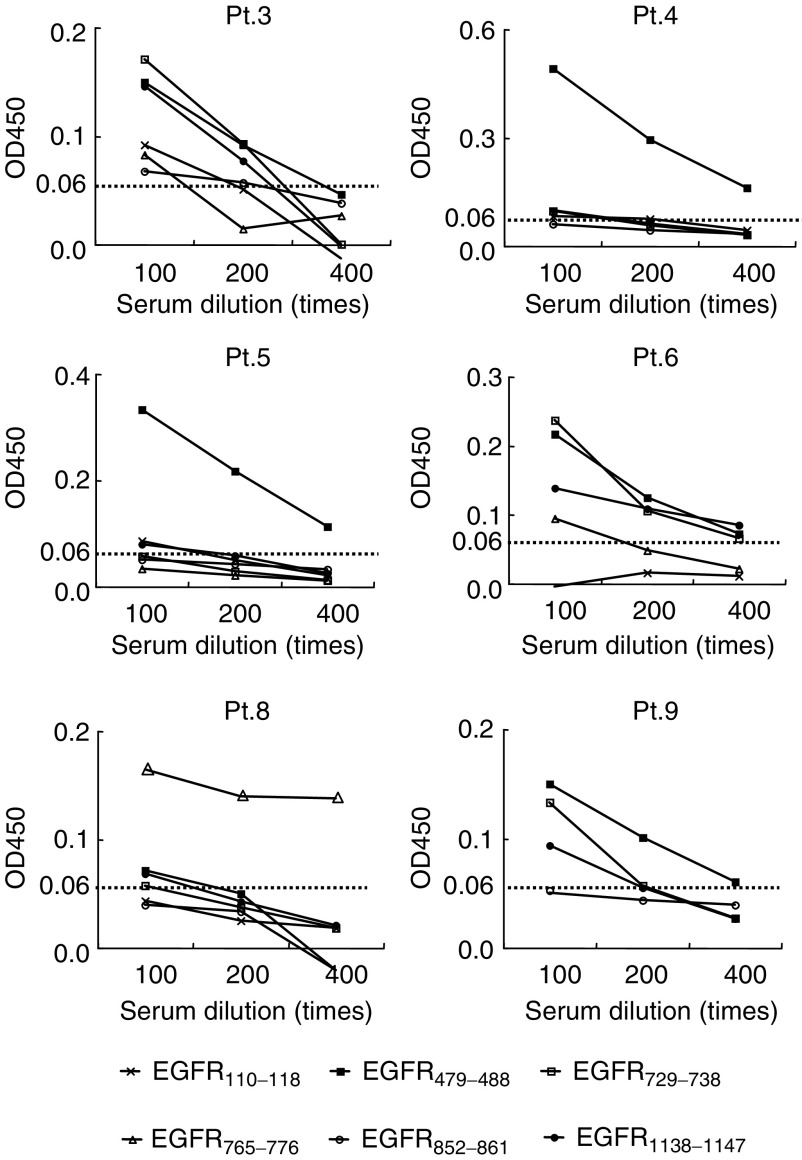
Detection of antipeptide IgG. Optical density values of each sample were assayed in serially diluted serum samples to estimate peptide-reactive IgG levels by the ELISA. The OD value against an irrelevant peptide (HIV) used as a negative control was subtracted from the data. Representative results of six patients (Pts.3, 4, 5, 6, 8, and 9) are shown. The cutoff value was set as 0.06 OD value at a serum dilution of 100 times (the mean (0.02) +2 s.d. (0.02) of OD value in HDs (*n*=11) in response to an HIV peptide which has an HLA-A2 binding motif taken as a negative control).

We first investigated whether IgG reactive to each of the 29 different EGFR-derived peptides could be detected in the sera of 20 cancer patients and 11 HDs. Representative results are shown in Figure 1

and a summary of the results on 11 different peptides to which at least two of the sera showed a positive response is given in Table 1

**Table 1:** Humoral responses to the EGFR peptides

**Responses to the EGFR peptides (OD values)^a^**													
**Subjects**	**HLA**	**Subtype**	**EGFR_10–18_**	**EGFR_61–70_**	**EGFR_110–118_**	**EGFR_479–488_**	**EGFR_599–607_**	**EGFR_653–662_**	**EGFR_654–662_**	**EGFR_729–738_**	**EGFR_765–776_**	**EGFR_852–861_**	**EGFR_1138–1147_**
Pt.1	A2/24	A0207	—b	—	—	—	—	—	—	—	—	—	—
Pt.2	A2/24	A0206	—	—	—	—	—	—	—	—	—	—	—
Pt.3	A2/24	A0206	—	—	0.09	0.15	—	—	—	0.17	0.08	0.07	0.15
Pt.4	A2/11	A0206	0.26	—	0.08	0.49	—	—	—	0.10	—	—	0.10
Pt.5	A2	A0201	—	—	0.09	0.33	—	—	—	—	—	—	0.08
Pt.6	A2/24	A0206	—	—	—	0.22	—	—	—	0.24	0.10	—	0.14
Pt.7	A2/3	A0201	—	—	—	—	—	—	—	—	—	—	—
Pt.8	A2/24	A0206	—	—	—	0.07	—	—	—	—	0.16	—	0.07
Pt.9	A2/24	A0201	—	—	—	0.15	—	—	—	0.13	—	—	0.09
Pt.10	A2	A0207	—	0.07	0.14	0.12	0.13	0.07	—	0.07	—	—	0.13
Pt.11	A24/33		—	0.07	—	0.10	0.17	—	0.09	0.17	—	—	0.16
Pt.12	A24		—	—	—	0.21	0.08	—	—	—	—	—	0.07
Pt.13	A24		—	—	—	—	0.07	—	—	—	—	—	—
Pt.14	A24		—	—	—	—	—	—	—	—	0.18	—	—
Pt.15	A24		—	—	—	0.55	—	—	—	0.59	—	—	—
Pt.16	A24		—	—	—	0.17	—	—	—	—	—	—	—
Pt.17	A24/31		—	—	—	0.24	—	—	—	0.10	—	0.07	0.10
Pt.18	A24		—	—	—	0.07	—	—	—	—	—	—	—
Pt.19	A24/33		—	—	—	—	—	1.22	—	0.31	—	—	—
Pt.20	A24/11		—	—	—	—	—	—	—	0.30	—	—	—
													
HD1	A2/24	A0206	—	—	—	—	0.20	—	0.07	—	—	—	0.13
HD2	A2/24	A0206	—	—	—	—	0.09	—	—	—	—	—	—
HD3	A2/11	A0206	—	—	—	0.26	—	—	—	—	—	—	—
HD4	A2/26	A0201	—	—	—	0.13	—	—	—	—	—	—	0.08
HD5	A2	A0206	—	—	—	—	—	—	—	—	—	—	—
HD6	A2/24	A0201	—	—	—	—	—	—	—	—	—	—	—
HD7	A24/33		—	—	—	—	—	—	—	—	—	—	—
HD8	A24/26		—	—	—	—	—	—	—	—	—	—	—
HD9	A24/26		0.07	0.07	—	—	0.20	—	0.12	—	—	—	0.26
HD10	A24		—	—	—	—	—	—	—	—	—	—	0.17
HD11	A11/33		—	0.09	0.09	—	0.13	—	0.07	—	—	—	0.15
Antipeptide Abs		Pt (*n*=20)	1	2	4	13	4	2	1	10	4	2	10
		HD(*n*=11)	1	2	1	2	4	0	3	0	0	0	5

a Antipeptide IgG was assayed by ELISA as described in Materials and methods. Values represent the OD value at a serum dilution of 100 times.

b The OD values lower than the cutoff (0.06) are shown as —.

. Significant levels of IgG (>0.06 OD values at a serum dilution of 1 : 100) reactive to the EGFR_479-488_, EGFR_729-738_, and EGFR_1138-1147_ peptides were detected in the sera of 13, 10, and 10 patients, respectively. Sera from 2, 0, and 5 out of 11 HDs tested also showed significant levels of IgG reactive to the EGFR_479-488_, EGFR_729-738_, and EGFR_1138-1147_ peptides, respectively. In addition, the significant levels of IgG reactive to the EGFR_110-118_, EGFR_599-607_, and EGFR_765-776_ peptides were detected in sera from each of four cancer patients as well as a few HDs. The IgG reactive to the other five peptides was also observed in the sera of several patients and HDs. These humoral responses to EGFR peptides were observed in both HLA-A2-positive and -negative subjects, indicating no apparent HLA-A2 restriction to peptide-reactive IgG as reported previously (Ohkouchi *et al*, 2002). In contrast, significant levels of IgG reactive to the remaining 21 peptides were not detectable in any of the sera tested (data not shown). Subsequently, we focused our efforts on the five peptides EGFR_110–118_, EGFR_479–488_, EGFR_729–738_, EGFR_852–861_, and EGF-R_1138–1147_ (>90% purity) in the following study.

**Figure 2:**
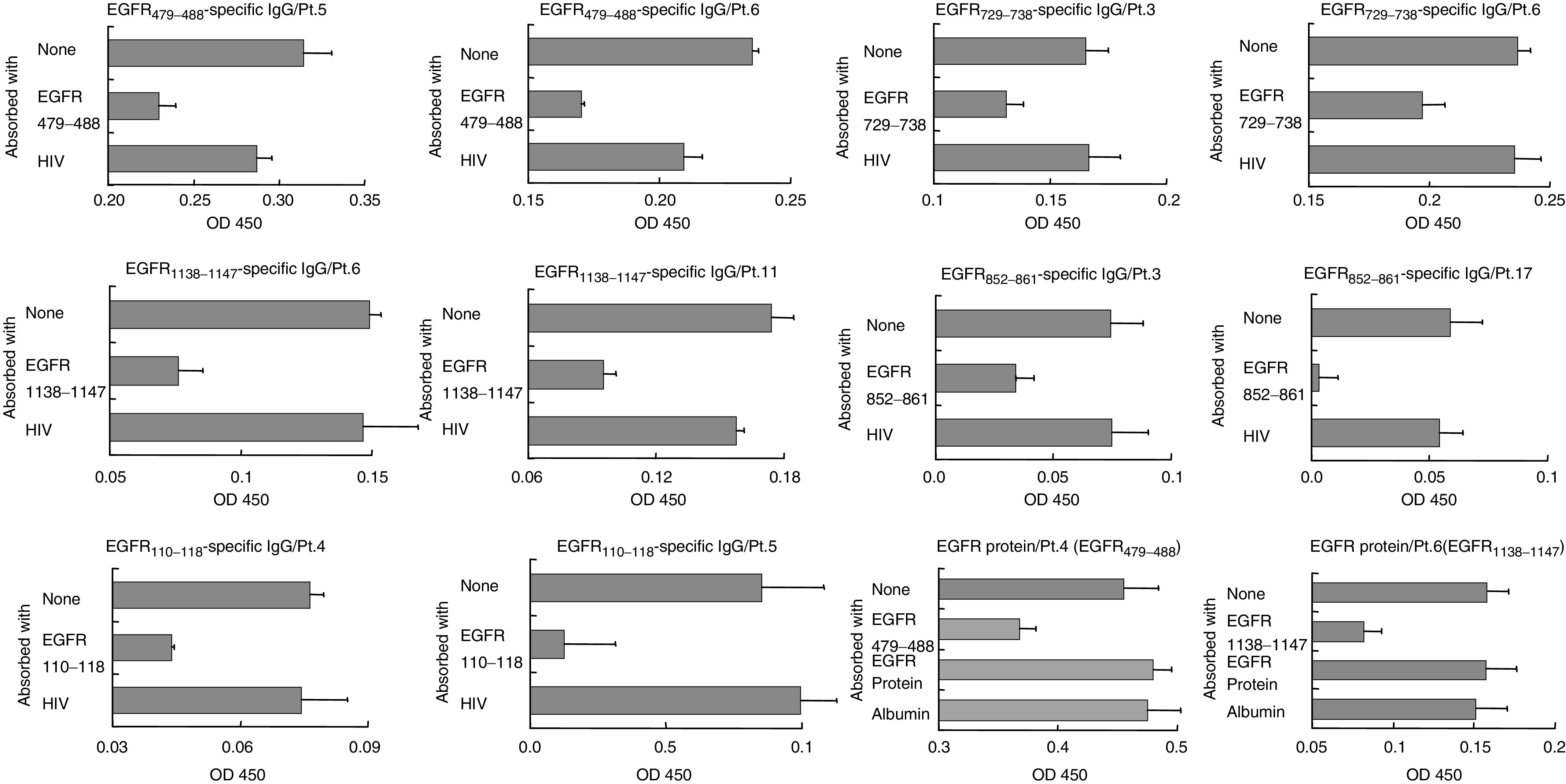
Specificity of antipeptide IgG. Each serum sample was absorbed with either a corresponding peptide or an HIV peptide used as a negative control three times at 37°C followed by testing of peptide-specific IgG activity with ELISA. Results of five peptides in the sera of each two representative patients from six (Pts. 3, 4, 5, 6, 11, and 17) are shown in the figure. In addition for testing of the peptide-specific IgG activity to whole protein, each serum sample was absorbed with an EGFR protein (purified from A431 cells), human albumin as a negative control, or a corresponding peptide as a positive control. The representative results from the sera of Pts.4 and 6 are shown in the lower, right-hand columns of this figure.

The peptide specificity of antipeptide IgG to each of the EGFR_110–118_, EGFR_479–488_, EGFR_729–738_, EGFR_852-861_, and EGF-R_1138-1147_ peptides was confirmed by absorption tests. Representative results of the peptide specificity for each of the five peptides by means of the absorption tests are shown in Figure 2

in which the results on sera from two patients were provided for each peptide. As can be seen, the activity of these sera reactive to each peptide was absorbed with a corresponding peptide, but not with an HIV peptide used as a negative control. We also investigated by an absorption test whether antipeptide IgG reacts to the whole EGFR protein. The level of the antipeptide IgG reactive to any of these peptides, however, was not decreased at all by the absorption test. Representative results of the two peptides (EGFR_479-488_ and EGFR_1138-1147_) are shown in the lower, right-hand columns of Figure 2. These results suggest that there was no crossreactivity between antipeptide IgGs and the whole EGFR protein.

**Figure 3:**
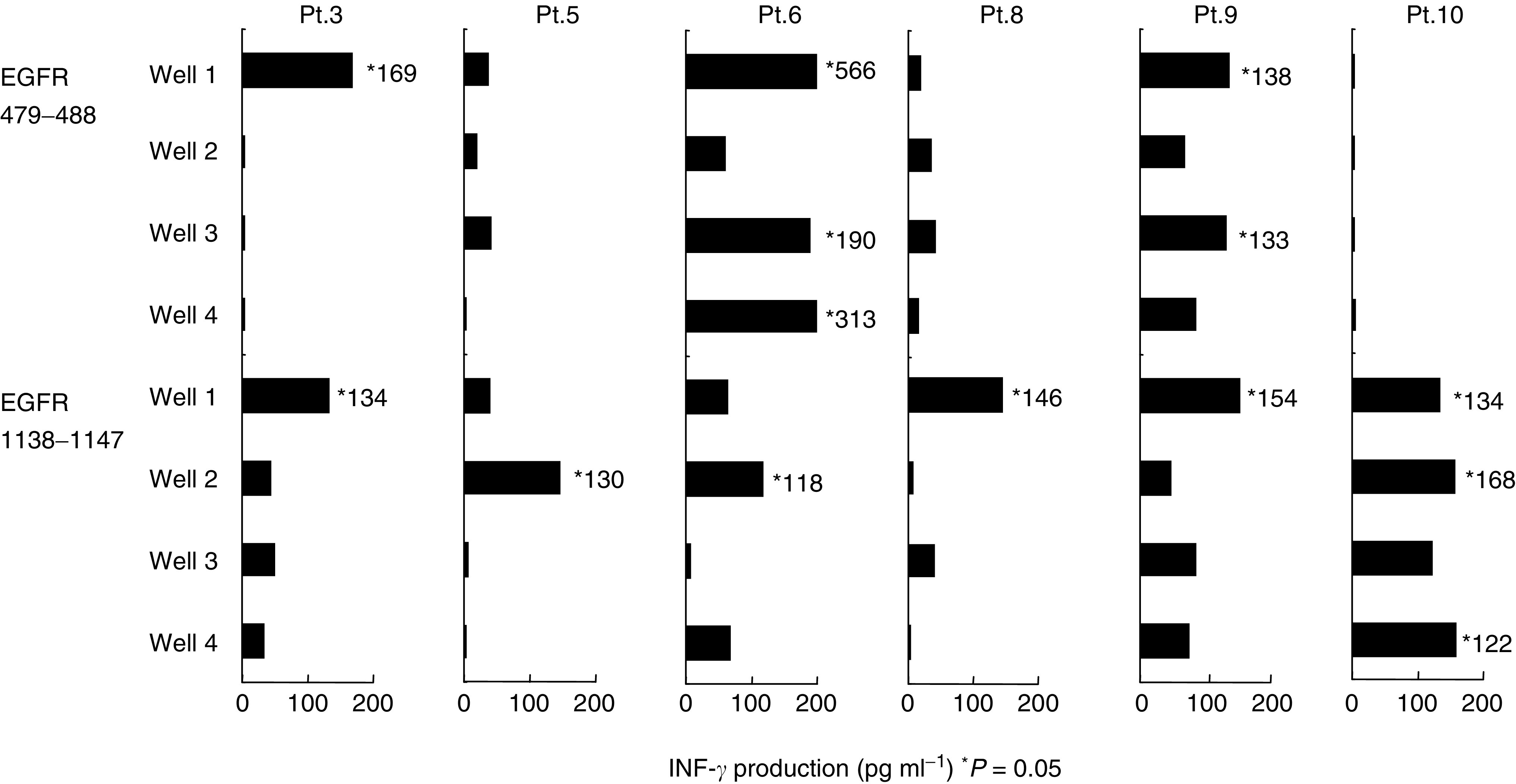
Cellular responses to peptide. Peptide-stimulated PBMCs from HLA-A2^+^ cancer patients were cultured in four different wells (15 × 10^4^ well^−1^). On day 14 of culture, the peptide-stimulated PBMCs (80−120 × 10^4^ well^−1^) from each well were independently collected and divided into four equal portions. Two such portions were separately tested for their ability to produce IFN-*γ* in response to T2 cells pulsed with a corresponding peptide, while the remaining two portions were tested with a negative control peptide (HIV). Background IFN-*γ* production in response to the HIV peptide (<50 pg ml^−1^) was subtracted. An asterisk (^*^) indicates *P*<0.05 by a two-tailed Student's *t*-test. The representative results of six patients (Pts.3, 5, 6, 8, 9, and 10) are shown.

Based on these findings, these EGFR_110–118_, EGFR_479–488_, EGFR_599–607_, EGFR_729–738_, EGFR_765–776_, and EGFR_1138–1147_ peptides were further tested for their abilities to induce CTL activity in PBMCs of HLA-A2^+^ epithelial cancer patients and HDs (*n*=10 and 6). The EGFR_813–822_ peptide, to which no IgG response was detectable in sera, was also tested as a control. We judged the induction to be successful when the supernatant of at least one well showed more than 100 pg ml^−1^ INF-*γ* production with a statistically significant difference (*P-*value of <0.05). The EGFR_479–488_ and EGF-R_1138–1147_ peptides induced peptide-specific CTLs in three and six of 10 cancer patients tested, respectively. Representative results (Pts.3, 5, 6, 8, 9, and 10) are shown in Figure 3

, in which the results from each of the four wells are provided. Background INF-*γ* productions in response to an HIV peptide (<50 pg ml^−1^) were subtracted. In regard to HLA-A subtypes, two (Pts.5 and 9), three (Pts.3, 6, and 8), and one (Pt.10) patients were HLA-A0201, -A0206, and -A0207, respectively (Table 1). The results indicate that these two peptides had the ability to induce a peptide-specific cellular response in PBMCs from different HLA-A2 subtypes. These two peptides, however, were not sufficiently stimulated to produce significant levels of IFN-*γ* in any of the six HDs tested. Similarly, each of the other five peptides tested rarely stimulated PBMCs to produce the significant levels of IFN-*γ* in either cancer patients or HDs (data not shown).

**Figure 4:**
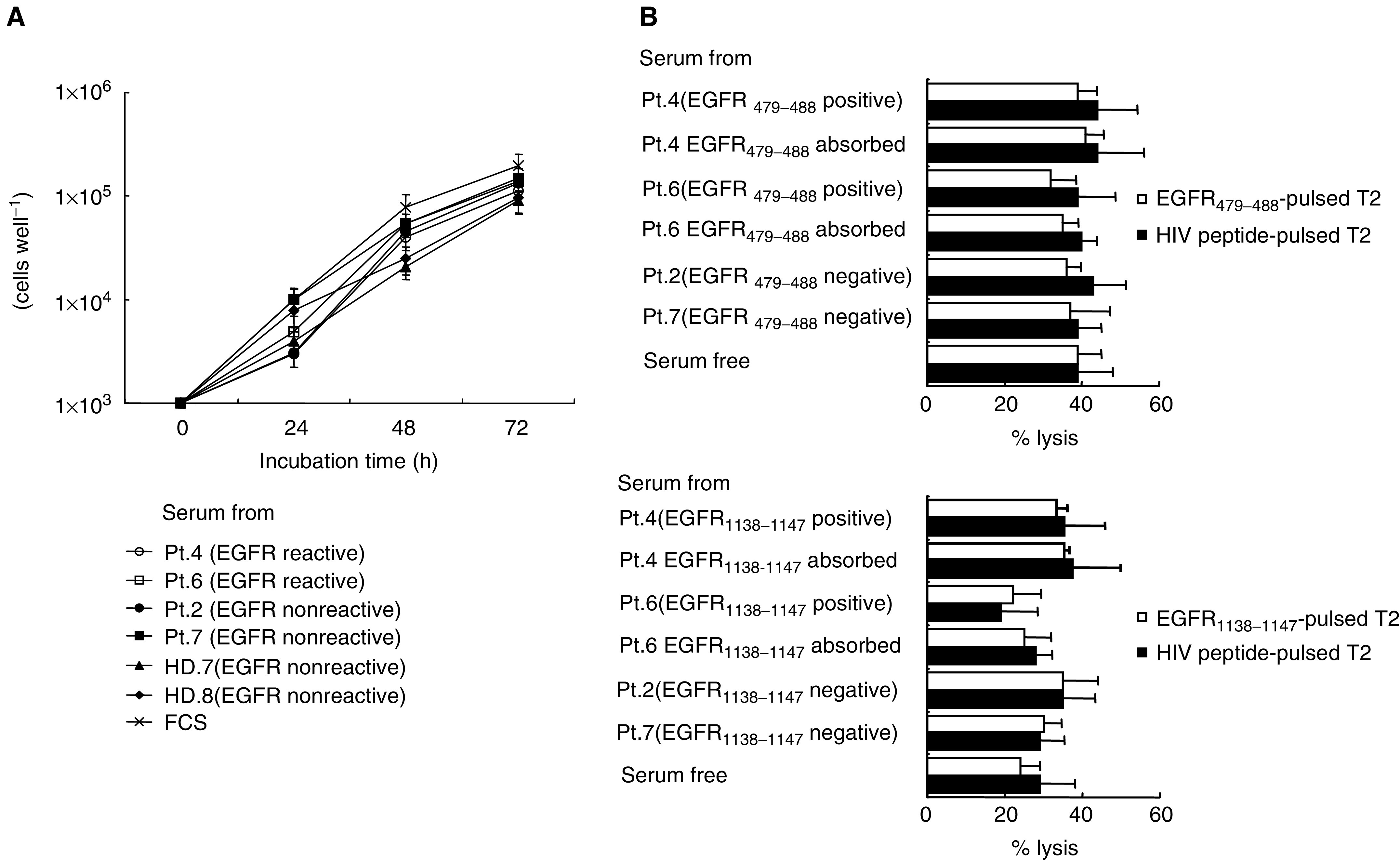
Direct inhibition and antibody-dependent cell-mediated cytotoxicity of sera possessing antipeptide IgG. (**A**) To test the direct growth inhibition activity of antipeptide IgG, 11–18 tumour cells were cultured in the presence of three different concentrations of sera, which had detectable levels of antipeptide activity. The 11–18 cells at 1 × 10^3^ cells well^−1^ in a 96-well microculture plate (IWAKI, Chiba, Japan) were cultured for 12 h in the medium with 10% FCS followed by replacement of the culture medium to serum-free RPMI1640 with 1, 2, or 5% of serum possessing antipeptide activity. As controls, the same volumes of these sera absorbed with a corresponding peptide as well as sera possessing no antipeptide activity were used for the culture. The 11–18 cells were also cultured in the RPMI with 1, 2, and 5% FCS, respectively. After 24, 48, and 72 h incubation, the number of viable cells was determined by Cell Counting Kit-8 (Dojindo, Kumamoto, Japan). The values are the mean±s.e. of quadruplicate cultures. (**B**) To test the antibody-dependent cell-mediated cytotoxicity of antipeptide IgG, the cytotoxicity of freshly isolated PBMCs from HLA-A2^+^ HDs against T2 cells pulsed with an EGFR-derived peptide or an HIV peptide as a negative control was measured in the presence of heat-inactivated serum possessing antipeptide activity by a standard 6-h ^51^Cr-release assay. As controls, these sera absorbed with a corresponding peptide, sera possessing nonpeptide activity, and serum-free RPMI1640 medium were used. All sera used in the cytotoxicity assay were heat-inactivated at 56^°^C in 30 min. The standard 6-h ^51^Cr-release assay was performed at three *E*/*T* (effector to target) ratios. The representative results on EGFR_479–488_ and EGFR_1138–1147_ peptides are shown in this figure (left side). The results were performed at *E*/*T* ratio 10 : 1, and the values represent the mean±s.d. of % specific lysis in triplicate assays.

Sera possessing anti- EGFR_479–488_ and anti-EGFR_1138–1147_ peptide activities from the patient 4 and patient 6 were tested for their capability to directly inhibit growth of 11–18 tumour cells. As controls, these sera absorbed with a corresponding peptide, sera without antipeptide activity from the two patients and two HDs, and FCS were used. However, none of the sera with antipeptide IgG directly inhibited tumour cell growth *in vitro*. Representative results at a serum concentration of 5% are shown in Figure 4

(left side). We also tested the antibody-dependent cell-mediated cytotoxicity of antipeptide IgG. Namely, the cytotoxicity of freshly isolated PBMCs from HLA-A2^+^ HDs against T2 cell pulsed with an EGFR-derived peptide or an HIV peptide as a negative control was measured in the presence of heat-inactivated serum possessing antipeptide activity. As controls, these sera absorbed with a corresponding peptide, sera possessing nonpeptide activity, and serum-free RPMI1640 medium were used. However, the presence of sera possessing anti-EGFR peptide activity could not increase their cytotoxicity. Representative results are shown in Figure 4 (right side).

**Figure 5:**
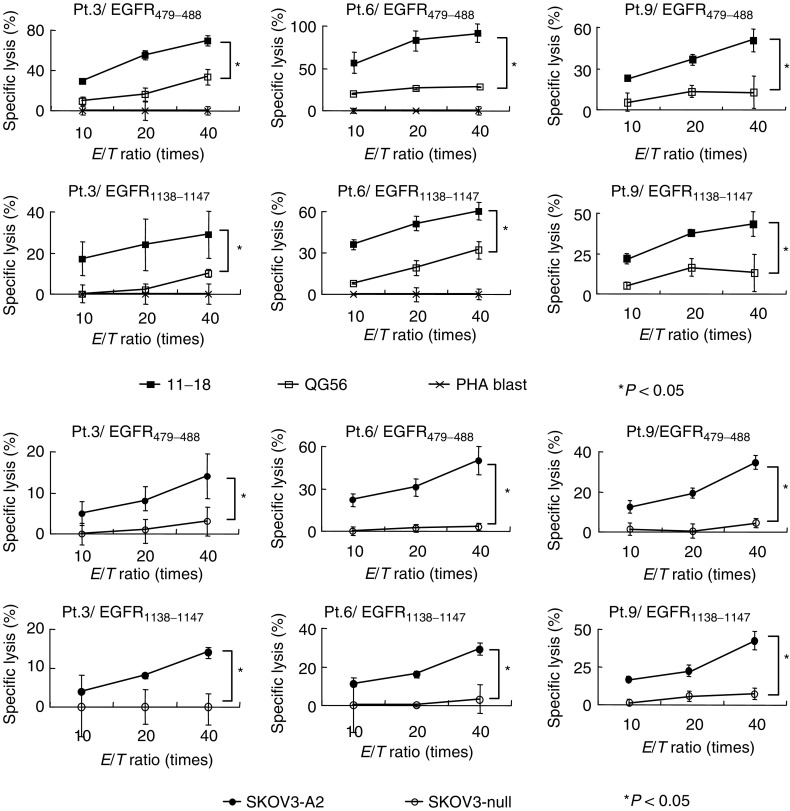
Cytotoxicity. Peptide-stimulated PBMCs were tested for their cytotoxicity against the following cancer cell lines: 11–18 (HLA-A2^+^, EGFR^+^), QG56 (HLA-A2^−^, EGFR^+^), SKOV3-A2 (HLA-A2^+^, EGFR^+^), and SKOV3 (HLA-A2^−^, EGFR^+^). PHA-blastoid T cells (HLA-A2^+^, EGFR^−^) were also used as a negative control. The standard 6-h ^51^Cr-release assay was performed at three *E*/*T* (effector to target) ratios. The representative results of three cancer patients (Pts.3, 6, and 9) are shown in the figure. Values represent the mean±s.d. of % specific lysis. An asterisk (^*^) indicates *P*<0.05 by a two-tailed Student's *t*-test.

The cytotoxicity of the EGFR_479–488_ or EGF-R_1138–1147_ peptide-stimulated PBMCs was confirmed by a 6-h ^51^Cr-release assay, and the representative results of the three patients (Pts.3, 6, and 9) are shown in Figure 5

. These PBMCs showed significant levels of cytotoxicity against all the 11–18 cells (HLA-A2^+^, EGFR^+^) and SKOV3-A2 cells (HLA-A2^+^, EGFR^+^), but failed to kill any of the QG56 cells (HLA-A26, EGFR^+^) or SKOV3 cells (HLA-A3/28, EGFR^+^) tested. These PBMCs also failed to kill PHA-blastoid T cells (HLA-A2^+^, EGFR^−^). Peripheral blood mononuclear cells stimulated with an HIV peptide taken as a negative control did not show such HLA-A2-restricted cytotoxicity (data not shown). These results suggest that these PBMCs possess HLA-A2-restricted cytotoxicity reactive to EGFR^+^ tumou cells.

**Figure 6:**
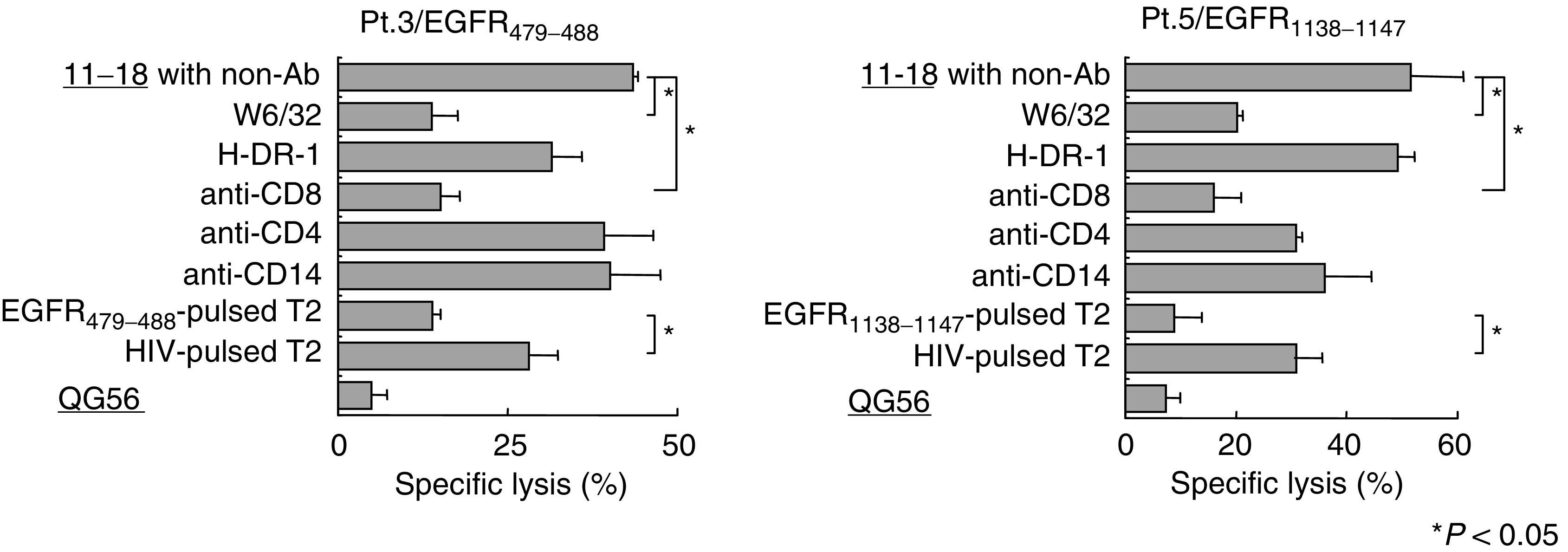
Inhibition and competition assays. Peptide-stimulated PBMCs were tested for their restriction and peptide-specificity of cytotoxicity against 11–18 (HLA-A2^+^, EGFR^+^) and QG56 (HLA-A2^−^, EGFR^+^) by the standard 6-h ^51^Cr-release assay. In all, 20 *μ*g/ml of anti-HLA-class I (W6/32, IgG2a), anti-HLA-class II (H-DR-1, IgG2a), anti-CD8 (Nu-Ts/c, IgG2a), and anti-CD4 (Nu-Th/i, IgG1) mAb were used for the inhibition assays. Anti-CD14 (JML-H14, IgG2a) mAb served as a negative control. For the competition assay, unlabelled T2 cells pulsed with the corresponding peptide or an HIV peptide as a negative control were added to the ^51^Cr-release assay at a cold-to-hot target cell ratio of 10 : 1. The 6-h ^51^Cr-release assay was performed at an *E*/*T* ratio of 10 : 1. An asterisk (^*^) indicates *P*<0.05 by a two-tailed Student's *t*-test. Values represent the mean±s.d. of % specific lysis.

Further, the restriction and peptide-specificity of the cytotoxicity were confirmed by inhibition and competition assays, respectively (Figure 6

). Namely, levels of the cytotoxicity mediated by these peptide-stimulated PBMCs were significantly inhibited by anti-HLA-class I (W6/32) or anti-CD8 mAb, but not by the other mAbs tested in the assay. The cytotoxicity was also inhibited by the addition of the corresponding peptide-pulsed T2 cells, but not by addition of the HIV peptide-pulsed cells. These results suggest that the CTL activity is largely mediated by the peptide-specific CD8^+^ T cells in an HLA-class I-restricted manner.

## DISCUSSION

Among the 29 EGFR-derived peptides tested in this study, two peptides, one at position 479–488 and the other at position 1138–1147, were recognised by cellular and humoral immune responses in at least one-third of PBMCs and half of the sera samples from HLA-A2^+^epithelial cancer patients, respectively. These peptides, however, rarely induced CTL activity in the PBMCs of HDs, although IgG reactive to them was detectable in the sera of some HDs. The reactivity of PBMCs from several of HDs to EGFR peptides is not particularly surprising, given that EGFR is expressed not only in epithelial cancer cells but also in certain normal epithelial cells (Coussens *et al*, 1985; Yamamoto *et al*, 1986; Salomon *et al*, 1995). Humoral responses to EGFR in sera of patients with different malignancies were reported (Bei *et al*, 1999). Aberrant expression and activation of EGFR in malignant cells might lead to breakdown of immunotolerance. Cellular responses to HER2/neu-derived peptides are also detectable in PBMCs from both cancer patients and HDs, whereas humoral responses to those HER2/neu peptides were not reported (Fisk *et al*, 1995; Peoples *et al*, 1995; Kawashima *et al*, 1999; Okugawa *et al*, 2000). It is of note, however, that at least one-third of PBMCs and sera samples from epithelial cancer patients in the present study showed both cellular and humoral responses to these two peptides, suggesting that these peptides have higher immunogenecity than any of the remaining 27 EGFR-derived peptides, which triggered immune responses only in a few subjects.

In addition to these two peptides, the four peptides, to which antipeptide IgGs were detectable in the sera of some cancer patients, were tested for their ability to induce peptide-reactive IFN-*γ* production in several cancer patients, but none of the four peptides induced the CTL activity under employed conditions. Among the four peptides, the EGFR_729–738_ peptide was recognised by the majority of patients' sera, but by none of the sera of the HDs, suggesting that CD4^+^ T cells of cancer patients may be involved in the antipeptide-specific IgG production. This point needs to be further studied in order to develop a monoclonal antibody to this epitope. Cellular responses to the remaining 23 peptides with HLA-A2 binding motifs were not investigated because of the limited number of PBMCs available for the analysis. Therefore, further studies will be needed to identify the EGFR-derived peptides capable of inducing HLA-A2-restricted cellular response alone.

We previously reported that IgG reactive against CTL epitope peptides was often detected in the prevaccination sera of cancer patients and also in the sera of HDs, and there was no obvious HLA-class I-A restriction involved (Ohkouchi *et al*, 2002; Kawamoto *et al*, 2003; Mine *et al*, 2003; Noguchi *et al*, 2003; Sato *et al*, 2003). Further, some CTL-directed peptides have shown the ability to elicit both cellular and humoral immune responses *in vivo* in phase I clinical studies, and levels of antipeptide IgG in postvaccination sera have well correlated with the overall survival of advanced cancer patients who received peptide vaccination (Mine *et al*, 2003; Sato *et al*, 2003). In contrast, IgG reactive to these CTL peptides has been reported to be either lacking or unbalanced in the sera of patients with atopic disease (Kawamoto *et al*, 2003). These results suggest that the IgG to these peptides play a role in host-defence against these diseases, although the underlying mechanism of the antitumour immune responses in cancer patients is presently unclear. The underlying mechanisms of IgG production against CTL epitope peptides in HDs as well as the disturbance of IgG production in patients with atopic disease are also presently unclear. Those antipeptide IgGs, however, have not been found to react to the mother proteins to the degree that they have been tested, which is in agreement with the present finding of a lack of anti-EGFR-derived-peptide IgG reactivity in response to the EGFR protein shown. Sera possessing anti-EGFR-derived-peptide IgGs also failed to show either direct growth inhibition of tumour cells *in vitro* or to elicit antibody-dependent cell-mediated cytotoxicity to tumour cells to the degree that they have been tested. Therefore, anti-EGFR-derived-peptide IgGs may not act directly on tumour cells.

These antipeptide IgGs did not react to the mother protein, and also failed to show either the direct inhibition of tumour cell growth *in vitro* or to elicit antibody-dependent cell-mediated cytotoxicity to tumour cells as far as tested. It is well known that T cells in the circulation rarely infiltrate into tumour sites. In contrast, IgG molecules might easily reach either peritumour sites or intratumour sites, which in turn facilitate inflammatory reactions at the tumour cites. This assumption is in part supported by the recent observation that significant levels of inflammatory responses were observed around prostate cancers at the time of surgery in patients who received peptide vaccinations based on information regarding antibodies reactive to peptides before radical prostatectomy (Noguchi *et al* unpublished results). We also reported that IgG reactive to these CTL-epitope peptides are either lacking or unbalanced in the sera of patients with atopic disease (Kawamoto *et al*, 2003). The results shown in this study along with those from noncancerous subjects suggests that these peptide-reactive IgGs play a role in host-defence against various diseases, although further studies are needed to clarify their biological role as well as their mechanism of action.

Although further studies are needed to clarify the biological role as well as the mechanism of action of antipeptide antibodies, the two peptides recognised by both cellular and humoral immune responses can be presumed to be more immunogenic than those recognised by the cellular response alone. The HLA-A2 allele is found in 40% of Japanese, 50% of Caucasians, and 16% of Africans. There are several major subtypes of the HLA-A2 allele. The frequencies of HLA-A0201, -A0206, and -A0207 among HLA-A2-positive Japanese are about 45, 36, and 17%, respectively, whereas HLA-A0201 is the predominant subtype among HLA-A2-positive Western Caucasians (96%), African Blacks (62%), and Sardinian Caucasians (59%) (Imanishi *et al*, 1992). These two peptides at positions 479–488 and 1138–1147 possessed the ability to induce HLA-A2-restricted and tumour-specific CTLs from PBMCs of cancer patients with at least the three different HLA-A2 subtypes shown above.

Epidermal growth factor receptor is highly expressed in a number of human tumours (Coussens *et al*, 1985; Yamamoto *et al*, 1986; Salomon *et al*, 1995), and many clinical trials of EGFR-targeted therapies have been going on. In those clinical trials, various toxicities (mainly, acneiform rash and diarrhoea) were reported although the frequency and severity of these adverse events were relatively low (Dittrich *et al*, 2002; Herbst *et al*, 2002; Mendelsohn and Baselga, 2003). We have used self-antigen-derived peptides, such as SART1, SART3, and lck, in phase I clinical studies of individualised peptide vaccination for far advanced cancer patients. In these clinical studies, no severe adverse events, except for local redness and swelling of injection site, were observed (Mine *et al*, 2003; Noguchi *et al*, 2003; Sato *et al*, 2003; Tsuda *et al*, 2004). Epidermal growth factor receptor-derived peptides shown in this study are now under clinical trials in our hospitals as phase I study of individualised peptide vaccination for far advanced cancer patients, but the vaccination of these EGFR-peptides was not associated with acneiform rash and diarrhoea. However, careful observation throughout the phase I study is needed to obtain the safety of these peptides. The phase II study with these peptides is planned to see whether EGFR is one of attractive targets for immunotherapy for far advanced epithelial cancer patients or not.

In conclusion, these findings may provide new insight for the development of an EGFR-based immunotherapy beneficial for substantial numbers of epithelial cancer patients throughout the world.
